# Can atherogenic indices and the triglyceride-glucose index be used to predict erectile dysfunction?

**DOI:** 10.1093/sexmed/qfad069

**Published:** 2024-01-19

**Authors:** Murat Sambel, Abdullah Erdogan, Volkan Caglayan, Sinan Avci, Sahin Kilic, Halil Emre Yildiz, Ercument Keskin

**Affiliations:** Department of Urology, Antalya Training and Research Hospital, Varlik 07100, Antalya, Turkey; Department of Urology, Bursa City Hospital, Nilufer 16110, Bursa, Turkey; Department of Urology, Bursa City Hospital, Nilufer 16110, Bursa, Turkey; Department of Urology, Bursa City Hospital, Nilufer 16110, Bursa, Turkey; Department of Urology, Antalya Training and Research Hospital, Varlik 07100, Antalya, Turkey; Department of Urology, Mus State Hospital, 49100, Mus, Turkey; Department of Urology, Mengucek Gazi Education and Research Hospital, Erzincan Binali Yildirim University, 24002, Erzincan, Turkey

**Keywords:** atherogenic indices, erectile dysfunction, endothelial dysfunction, insulin resistance, triglyceride-glucose index

## Abstract

**Background:**

Atherosclerosis and insulin resistance play an important role in the development of erectile dysfunction (ED), and few studies have comprehensively evaluated more specific indicators like atherogenic indices and the triglyceride-glucose (TyG) index in the assessment of ED.

**Aim:**

This study aimed to reveal the role of atherogenic indices (atherogenic index of plasma [AIP], Castelli risk index-1/2 [CRI-1/2], and atherogenic coefficient [AC]) based on plasma lipid ratios, which have been used as more sensitive indicators of atherosclerosis in recent years, and the TyG index, a practical indicator of insulin resistance, in predicting vasculogenic ED.

**Methods:**

The study included a total of 199 patients who met the inclusion criteria and a total of 51 control subjects without ED complaints according to the International Index of Erectile Function (IIEF-5) scores (>21) between May 2021 and October 2022. For all participants, the demographic and biochemical parameters were evaluated, and atherogenic indices, namely CRI-1 (total cholesterol/high-density lipoprotein [HDL]), CRI-2 (LDL/HDL) AIP [log10(triglycerides/HDL)], and AC (non-HDL/HDL), as well as the TyG index [Ln {fasting triglycerides (mg/dL) × fasting glucose (mg/dL)/2}] were calculated.

**Outcomes:**

The TyG index, which is an indicator of insulin resistance, and atherogenic indices such as CRI-1, AIP, and AC were significantly associated with ED, and especially AIP and the TyG index seem to be more important in the evaluation of ED.

**Results:**

According to the univariate analysis, the patient group had significantly higher CRI-1 (5.3 ± 1.4 vs 4.7 ± 1.3; *P* = .005), AIP (0.31 ± 0.26 vs 0.13 ± 0.2; *P* < .001), AC (4.1 ± 1.4 vs 3.70 ± 1.2; *P* = .026), and TyG (9.16 ± 0.71 vs 8.77 ± 0.52; *P* < .001) values compared with the control group. In the correlation analysis, a significant negative correlation was found between the AIP and TyG index and the IIEF-5 scores (r^2^ = 0.120, *P* < .001 between AIP and IIEF-5; r^2^ = 0.091, *P* < .001 between TyG index and IIEF-5). The multivariate analysis revealed AIP and the TyG index as independent predictive factors for ED.

**Clinical Implications:**

The use of atherogenic indices and TyG index in daily urology practice can help physicians in the diagnosis and follow-up of ED.

**Strengths and Limitations:**

The lack of sex hormone–binding globulin and free testosterone levels represents a limitation of our study. Another limitation is that the severity of ED was determined using the IIEF-5 scores, rather than a more objective method, such as penile artery ultrasound.

**Conclusion:**

Atherogenic indices and the TyG index can be used as inexpensive and practical markers to predict the severity of arteriogenic ED.

## Introduction

Erectile dysfunction (ED) is a common condition in adult men, explained as the consistent or recurrent inability to achieve and/or maintain a penile erection sufficient for sexual intercourse.^1^ The prevalence of ED varies widely around the world, ranging from 1% to 10% in men under 40 years of age, and ED is affecting more than half of the male population between 40 and 80 years of age.^2^ It is estimated that 150 million men worldwide are affected by ED, which is a public health problem that can have a major impact on patients’ quality of life.^3^ Psychogenic, organic, and/or both factors can cause ED; however, organic factors are more common. Vasculogenic ED is the most common subgroup of organic ED, with other cases having neurogenic and hormonal origins.^4^ Diabetes mellitus, hypertension, hyperlipidemia, obesity, and atherosclerotic cardiovascular diseases (CVDs) are the common risk factors.^5^ Although ED occurs for multifactorial reasons, the underlying pathology is mostly the development of atherosclerosis, following endothelial dysfunction due to a decrease in nitric oxide synthesis and bioavailability.^6^ Vascular integrity is crucial for erectile function.^7^ Atherosclerosis damages vascular beds, and due to its systemic nature, it can be hypothesized that all vascular beds are affected, but the onset of symptoms is related to the artery size.^8^ Gupta et al^9^ reported that ED may be an initial symptom of atherosclerosis, which reduces cavernosal blood supply and leads to vasculogenic ED. Montorsi et al^10^ showed that the prevalence of ED was significantly higher in patients with coronary artery disease (CAD) compared with men with normal coronary arteries due to the much more evident atherosclerotic burden detected on angiography.

A disorder of lipoprotein metabolism, known as dyslipidemia, is an accepted risk factor for atherosclerosis and CVD. Elevated levels of total cholesterol, low-density lipoprotein cholesterol (LDL), and triglycerides can be used for the assessment of CVD risk.^11^ These biomarkers can also be used to predict ED because of their common risk factors and close pathophysiological association with CVD.^12^ Currently, rather than conventional lipid parameters, more reliable predictors based on plasma lipid level ratios, such as the atherogenic index of plasma (AIP), Castelli risk index 1/2 (CRI-1/2), and atherogenic coefficient (AC), are available for use in the prediction of atherosclerosis and CVD.^13^^,^^14^

Insulin resistance (IR) is another well-known risk factor that leads to endothelial damage and atherosclerosis. Earlier studies have shown an association between IR and CVD.^15^^,^^16^ IR plays a significant role in endothelial dysfunction, and previous studies have reported that IR is also a major risk factor for ED.^17^^,^^18^ Simental-Mendía et al^19^ defined a simple, reliable, and low-cost IR marker, called the triglyceride-glucose index (TyG), calculated using fasting triglyceride and fasting glucose. Recent studies have widely used the TyG index as a marker of IR and found it to be positively associated with a higher prevalence of CVD.^20^^,^^21^ There is also a significant correlation between the TyG index and microvascular damage.^22^ Based on the close relationship between atherosclerosis, CVD, and ED, in this study we aim to evaluate the association between ED and atherogenic indices based on lipoprotein ratios and the TyG index, which is a marker of IR. In this study, we also asked: can these biomarkers give an idea about the baseline ED severity before starting ED treatment in daily clinical practice?

## Methods

We designed a cross-sectional observation study between May 2021 and October 2022. A total of 250 volunteer participants between 25 and 69 years of age who met the criteria of the study protocol were included in the study. The study protocol was reviewed and approved by the Clinical Research Ethics Committee of Erzincan Binali Yildirim University (E-21 142 744-804.99-77 745). The study complied with the rules of the Declaration of Helsinki. Written informed consent was obtained from all participants. A total of 199 patients diagnosed with ED (International Index of Erectile Function [IIEF-5] <22) according to the validated Turkish version of the IIEF-5 at our urology outpatient clinic and 51 control subjects without ED (IIEF-5 ≥22) were enrolled in the study. The control group was selected from the patients who presented to our outpatient clinic with nonsexual complaints and met the inclusion criteria of the study.

The mean ages of the patient and control groups were similar. Both groups consisted of sexually active individuals who had a regular sexual life for at least 6 months. Patients with an endocrine disease other than type 2 diabetes mellitus (DM), a history of malignant disease, anemia, any psychiatric disease, a history of previous penile or pelvic surgery/trauma, or penile curvature/Peyronie’s disease; those currently receiving treatment for ED; and those with congestive heart failure, chronic renal failure, chronic hepatic failure, a history of any neurological disease, or diabetic neuropathy were excluded from the study. The patients were questioned for nocturnal erections and tumescence during masturbation to exclude psychogenic ED. In addition, patients with a rapid onset of ED were considered to have psychogenic ED and excluded from the study. All participants were informed about the study in detail, and their written consent was obtained.

A clinical evaluation was performed on all participants. The presence of DM, hypertension, chronic obstructive pulmonary disease, and CAD was recorded. An anthropometric assessment was also undertaken, which included the measurements of height, weight, and body mass index (BMI). BMI was calculated by dividing body weight in kilograms by height in meters squared.

Fasting venous blood samples (12 hours) were obtained from all participants in the morning between 8 am and 10 am. Fasting blood glucose, complete blood count, hemoglobin A1c, liver function, C-reactive protein, blood urea nitrogen, creatinine, sodium, potassium, triglyceride, high-density lipoprotein (HDL), LDL, very LDL, total cholesterol, total testosterone, follicle-stimulating hormone, luteinizing hormone, prolactin, and prostate-specific antigen levels were analyzed. Biochemical parameters were measured by a Beckman Coulter AU 5800 clinical chemistry analyzer and hematological parameters were measured by the Sysmex XN-2000 hematology autoanalyzer by using commercially available test kits. Follicle-stimulating hormone, luteinizing hormone, prolactin, prostate-specific antigen, and total testosterone levels were measured with commercial assay kits using an Advia Centaur XPT immunoassay system (Siemens Healthcare Diagnostics). The values of atherogenic ratios, namely CRI-1 (TC/HDL), CRI-2 (LDL/HDL), AIP [log10 (triglycerides/HDL)], and AC (non-HDL/HDL), were calculated. The TyG index was expressed on a logarithmic scale and calculated as follows: Ln [fasting triglycerides (mg/dL) × fasting glucose (mg/dL)/2].^19^

For statistical analyses, the IBM SPSS Statistics for Windows version 25.0 package program was used. The compliance of the data with the normal distribution curve was evaluated with the Shapiro-Wilk test. Continuous variables and categorical data were compared using the Student *t* test, Mann-Whitney *U* test, and chi-square test. A 1-way analysis of variance was used to make statistical comparisons between ED subgroups formed according to IIEF-5 scores. Pearson correlation analysis was used to determine if there was a correlation between IIEF-5 and AIP or the TyG index. A logistic regression multivariate analysis was undertaken to identify independent predictive factors for the ED diagnosis. The receiver-operating characteristic (ROC) curve was used to evaluate and compare the predictive ability of LDL, AIP, and the TyG index for the ED diagnosis. *P* < .05 was accepted as statistically significant.

## Results

A total of 250 patients were evaluated, and 199 were included in the study as the ED group and 51 as the control group according to their IIEF-5 scores. The demographic characteristics of the ED and control groups are shown in Table 1. There was no significant difference in age between the ED and control groups. The mean ages of the ED and control groups were 49.6 ± 9.0 years and 47.6 ± 9.2 years, respectively. A statistically significant difference was found between the groups regarding BMI, incidence of DM, and the International Prostate Symptom Score, which were all higher in the ED group (Table 1). All enrolled participants were married and sexually active.

**Table 1:** Demographic characteristics of the study groups.

	**Control group**	**ED group**	***P***
Age, y	47.6 ± 9.2	49.6 ± 9.0	.136
BMI, kg/m^2^	26.74 ± 2.6	28.35 ± 4.2	.009*^a^*
HT, n(%)			.320
Absent	46 (83.6)	151 (77.4)	
Present	9 (16.4)	44 (22.6)	
DM, n(%)			.001*^a^*
Absent	52 (94.5)	145 (74.4)	
Present	3 (5.5)	50 (25.6)	
COPD, n(%)			.154
Absent	55 (100)	188 (96.4)	
Present	0 (0)	7 (3.6)	
CAD, n(%)			.209
Absent	53 (96.4)	178 (91.3)	
Present	2 (3.2)	17 (8.7)	
IIEF-5 score	23.3 (22-25)	11.0 (5-21)	<.001*^a^*
IPSS score	4.3 (0-10)	8.8 (0-32)	<.001*^a^*

Values are mean ± SD or n (%).

Abbreviations: BMI, body mass index; CAD, coronary artery disease; COPD, chronic obstructive pulmonary disease; ED, erectile dysfunction; HT, hypertension; IIEF-5, International Index of Erectile Function; IPSS, International Prostate Symptom Score.

^a^ Values are statistically significant (*P* < .05).

The comparison of hematological, biochemical, and hormonal parameters according to the ED status is presented in Table 2. The ED group had significantly higher fasting blood glucose, C-reactive protein, hemoglobin A1c, triglyceride, LDL, very LDL, total cholesterol, CRI-1, AIP, AC, and TyG index values compared with the control group. There was no statistically significant difference between the ED group and the control group in terms of other hematological, biochemical, and hormonal parameters.

**Table 2:** Hematological, biochemical, and hormonal parameters of the study groups.

	**Control group**	**ED group**	***P***
FBG, mg/dL	91.96 ± 11.9	121.32 ± 29.5	.001*^a^*
ALT, U/L	26.78 ± 11.3	29.74 ± 16.8	.220
AST, U/L	23.87 ± 5.0	25.7 ± 9.8	.082
CRP, mg/L	3.95 ± 1.7	4.98 ± 6.6	.046*^a^*
HbA1c, %	5.38 ± 0.4	6.20 ± 1.7	.001*^a^*
BUN, mg/dL	13.95 ± 3.6	14.2 ± 3.9	.152
Creatinine, mg/dL	0.85 ± 0.09	0.88 ± 0.17	.341
Sodium, mmol/L	140 ± 3.9	139.7 ± 10.2	.822
Potassium, mmol/L	4.42 ± 0.7	4.51 ± 0.3	.176
TG, mg/dL	161.7 ± 95.3	197.2 ± 110.1	.031*^a^*
HDL, mg/dL	42.74 ± 8.2	41.30 ± 9.7	.318
LDL, mg/dL	116.06 ± 23.2	129.06 ± 32.7	.001*^a^*
VLDL, mg/dL	30.56 ± 18.0	36.9 ± 21.8	.049*^a^*
Total cholesterol, mg/dL	193.3 ± 40.3	206.4 ± 40.1	.034*^a^*
Total testosterone, ng/dL	395.26 ± 125.59	382.01 ± 173.53	.608
FSH, mIU/mL	6.8 ± 2.1	7.3 ± 3.3	.184
LH, mIU/mL	4.8 ± 1.8	5.2 ± 2.1	.076
Prolactin, ng/mL	8.4 ± 2.9	8.8 ± 3.8	.553
HGB, g/dL	15.0 ± 1.3	15.0 ± 1.2	.932
WBC (10^3^/μL)	8.0 ± 2.0	8.5 ± 3.2	.659
PLT (10^3^/μL)	239.9 ± 49.6	250.0 ± 58.3	.243
PSA, ng/mL	0.94 ± 0.6	0.97 ± 0.7	.811
CRI-1	4.7 ± 1.3	5.3 ± 1.4	.005*^a^*
CRI-2	3.07 ± 1.2	3.08 ± 0.9	.903
AIP	0.13 ± 0.2	0.31 ± 0.26	<.001*^a^*
AC	3.70 ± 1.2	4.1 ± 1.4	.026*^a^*
TyG index	8.77 ± 0.52	9.16 ± 0.71	<.001*^a^*

Values are mean ± SD.

Abbreviations: AC, atherogenic coefficient; AIP, atherogenic index of plasma; ALT, alanine transaminase; AST, aspartate transaminase; BUN, blood urea nitrogen; CRI, Castelli risk index; CRP, C-reactive protein; ED, erectile dysfunction; FBG, fasting blood glucose; FSH, follicle stimulating hormone; HbA1c, hemoglobin A1c; HDL, high-density lipoprotein; HGB, hemoglobin; LDL, low-density lipoprotein; LH, luteinizing hormone; PLT, platelet; PSA, prostate-specific antigen; TG, triglyceride; TyG, triglyceride-glucose; VLDL, very low-density lipoprotein; WBC, white blood cell.

^a^ Values are statistically significant (*P* < .05).

**Figure 1:**
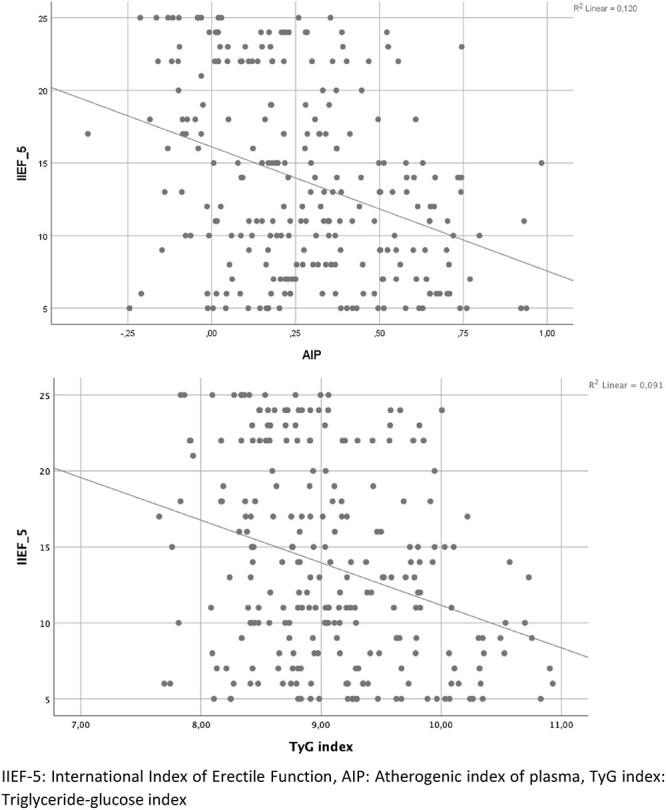
Results of the correlation analysis between International Index of Erectile Function (IIEF-5) and atherogenic index of plasma (AIP) and the triglyceride-glucose (TyG) index.

**Figure 2:**
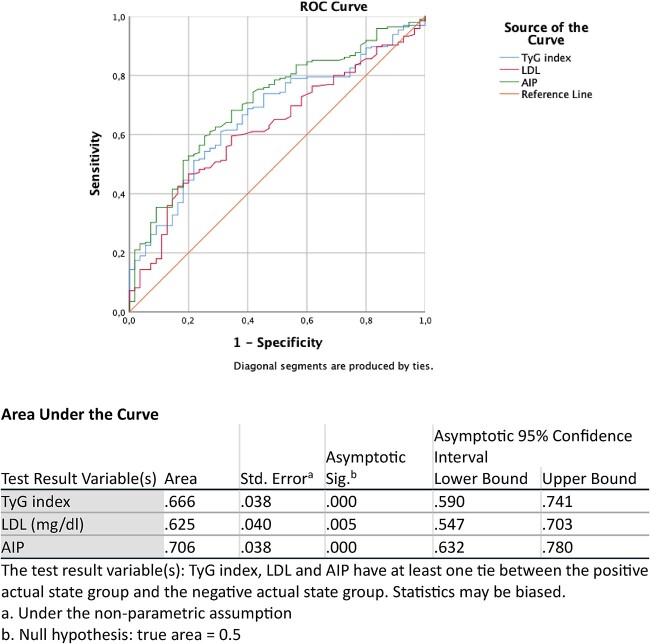
Receiver-operating characteristic (ROC) curves and area under the curve values of the investigated parameters.

In Pearson correlation analysis, IIEF-5 had a significant negative correlation with AIP (r^2^ = 0.120, *P <* .001) and the TyG index (r^2^ = 0.091, *P* < .001) (Figure 1). Table 3 shows the results of the multivariate logistic regression analysis performed between clinical variables and IIEF-5. In this analysis, LDL, AIP, and the TyG index were found to be independent predictors of ED. The ability of these parameters to predict the presence of ED was evaluated using ROC curve analysis, and the area under the curve values obtained are presented in Figure 2. It was determined that the TyG index, LDL, and AIP statistically significantly predicted the presence of ED. When the cutoff value of AIP was taken as 0.232, it had a sensitivity of 59% and a specificity of 74%. At a cutoff value of 8.91, the TyG index had 61% sensitivity and 69% specificity.

**Table 3:** Results of the multivariate logistic regression analysis between clinical variables and IIEF-5.

**Multivariate model**	**OR**	**95% CI**	***P***
LDL, mg/dL	1.027	1.009-1.052	.005*^a^*
AIP	33.171	14.965-45.225	<.001*^a^*
TyG index	0.014	0.001-0.247	.004*^a^*

Abbreviations: AIP, atherogenic index of plasma; CI, confidence interval; IIEF-5, International Index of Erectile Function; LDL, low-density lipoprotein; OR, odds ratio; TyG, triglyceride-glucose.

^a^ Values are statistically significant (*P* < .05).

A subgroup analysis was performed on the ED patients according to IIEF-5 scores in terms of CRI-1, CRI-2, AIP, AC, and the TyG index. As shown in Table 4, according to a 1-way analysis of variance test, CRI-1, AIP, and the TyG index showed significant differences between the ED subgroups. No statistically significant difference was found between the groups for CRI-2.

**Table 4:** Comparison between ED subgroups.

**ED subgroups according to IIEF-5 scores**	**CRI-1**	**CRI-2**	**AIP**	**AC**	**TyG index**
1 2 3 4	.882 .523 .006*^a^*	1.000 1.000 1.000	.009*^a^* .018*^a^* .003*^a^*	.457 .291 .200	.148 .090 .038*^a^*
2 1 3 4	.882 1.000 .409	1.000 1.000 1.000	.009*^a^* 1.000 1.000	.457 1.000 1.000	.148 1.000 1.000
3 1 2 4	.523 1.000 .587	1.000 1.000 1.000	.018*^a^* 1.000 1.000	.291 1.000 1.000	.090 1.000 1.000
4 1 2 3	.006*^a^* .409 .587	1.000 1.000 1.000	.003*^a^* 1.000 1.000	.200 1.000 1.000	.038*^a^* 1.000 1.000

Values are *P* values.

All statistical comparisons were performed using the analysis of variance to compare multiple group means: 1 indicates mild ED^17-21^; 2 indicates mild-to-moderate ED^12-16^; 3 indicates moderate ED^8-11^; 4 indicates severe ED.^5-7^

Abbreviations: AC, atherogenic coefficient; AIP, atherogenic index of plasma; CRI, Castelli risk index; ED, erectile dysfunction; IIEF-5, International Index of Erectile Function; TyG, triglyceride-glucose.

^a^ Significant results (*P* < .05).

## Discussion

ED is not a life-threatening disease, but it significantly affects the quality of life of the patient as well as that of his partner. The etiology of ED is generally categorized as organic, psychogenic, or mixed. Organic causes are responsible for 80% of cases and include vascular, neurogenic, hormonal, drug-related, or iatrogenic factors.^23^ The major cause of ED among aging men is organic disease due to vascular disruption caused by atherosclerosis.^24^ ED and atherosclerosis share the common risk factors of hypertension, dyslipidemia, DM, smoking, and obesity.^24^ ED is an early sign of a systemic disorder that can result in CAD. Penile arteries tend to be affected by atherosclerosis earlier than coronary arteries due to their small diameters.^10^

Some parameters used to predict CVD and atherosclerosis are also used for ED due to their similar risk factors, but more reliable markers have been developed for ED in recent years. In this study, we investigated whether some parameters that were recently introduced into clinical use as more reliable options than classical lipid parameters, according to previous studies, could be used to predict ED. Many studies have shown that CRI-1, CRI-2, AIP, and AC, known as atherogenic indices or lipid ratios, and the TyG index, which is a practical indicator of IR, have been shown to be more useful than conventional lipid parameters.^25-28^ Various studies conducted in recent years^29-31^ have demonstrated the relationship between ED and these indices evaluated individually. However, our study contributes to the literature by addressing the relationship of all these parameters with ED within the same article.

The lipid ratios (CRI-1, AIP, and AC) and the TyG index were found to be significantly higher in the ED group in the univariate analysis, and LDL, AIP, and the TyG index were shown to be independent predictive factors for ED in the multivariate analysis. In our study, in addition to atherogenic indices, LDL alone was determined to be an important parameter and presented as an independent predictive factor for ED. Hyperlipidemia may cause ED by affecting the vascular endothelial and smooth muscle cells of the penis. LDL is an important marker for ED and has also been shown to be a causative factor for the impaired relaxation response of the corpus cavernosum.^32^

Millan et al^33^ suggested that the CRI-1 and CRI-2 ratios were more valuable than conventional lipid parameters in terms of CVD risk. In addition, Bhardwaj et al^26^ emphasized that the CRI-1, CRI-2, and AC indices could be used as important parameters in predicting the risk of CVD and the development of atherosclerosis. In another study, CRI-1, CRI-2, and AC were found to be more delicate risk predictors for atherosclerotic CVD.^34^ In light of this literature, we evaluated the relationship between these indices and arteriogenic ED by considering the relationship of these indices with atherosclerosis. According to our univariate analysis, the CRI-1 and AC ratios were significantly higher in the ED group, but there was no significant difference between the ED and control groups in terms of CRI-2. In the multivariate analysis, none of these indices was an independent factor for ED. In a study conducted by Culha et al^30^ investigating the relationship between atherogenic indices and ED, the severity of ED was found to be significantly correlated with CRI-1 and AIP. This supports the findings of our study.

AIP, which is another reliable lipid ratio that can be obtained from a simple test, is accepted as an important predictor of CVD risk.^35^ AIP has been shown to be an important predictor of atherosclerosis, and AIP values above 0.24 have been associated with high CVD risk.^26^ Ermis et al,^29^ who evaluated the correlation of AIP with ED, concluded that AIP could be used as a useful marker to determine the severity of ED, as well as the severity of atherosclerosis. In the current study, the correlation analysis performed revealed a significant inverse correlation between AIP and IIEF-5, and the multivariate analysis showed AIP as an independent predictive factor for ED. In the ROC analysis, AIP values above 0.232 were associated with a higher ED risk. The results of our study are consistent with those of previous studies showing the relationship between AIP and ED severity.^29^^,^^30^

The TyG index is an inexpensive and reliable parameter to show IR, and it has been reported to be closely related to CVD and atherosclerosis in many studies conducted in recent years. For example, in a study by Park et al,^28^ a high TyG index was shown to be independently associated with the progression of coronary artery calcification, independent of other conventional CVD risk factors. Similarly, Da Silva et al^21^ showed that the TyG index was positively associated with a high prevalence of symptomatic CAD. In another study, a higher TyG index was associated with an increased risk of major cardiac and cerebrovascular events.^36^ These findings suggest that the TyG index can be used to predict atherosclerosis in the presence of CVD.^27^ In addition, Zhao et al^22^ showed that a high TyG index was significantly associated with a higher risk of arterial stiffness and nephric microvascular injury. The TyG index is also a valuable biomarker for the development of diabetes and has been shown to be associated with the incidence of this disease.^37^ In our study, the TyG index was significantly higher in the ED group, which also had a higher incidence of DM. We aimed to determine whether ED was related to the TyG index, which can be used as a marker of atherosclerosis and vascular damage and as an indicator of IR. In the correlation analysis performed, there was an inverse significant correlation between the IIEF-5 score and the TyG index, with the latter being found to be an independent predictive factor for ED in the multivariate analysis. Our results are consistent with those reported by Yılmaz et al,^31^ who also investigated the relationship between ED and the TyG index. Moreover, when the cutoff value of the TyG index was taken as 8.91 in our ROC analysis, it predicted ED with a sensitivity of 61% and a specificity of 69%. We consider that this cutoff value determined for the TyG index makes a significant contribution to the literature.

In this study, we also evaluated the ED subgroups formed according to the IIEF-5 scores separately with respect to each of the atherogenic indices and the TyG index (Table 4). When all ED subgroups were examined, there was a significant difference only between groups 1 and 4 (mild and severe ED groups) for CRI-1 and the TyG index, while a significant difference was found between all subgroups for AIP. Our study supports that these markers may be useful in predicting the severity of ED. As it is known, the relationship between the severity of ED and the mortality of CVDs has been revealed by various meta-analyses.^38^^,^^39^ ED may also be a precursor of underlying CVD, and having information about the severity of ED will also help to identify patients who need intensive counseling on lifestyle habits that may prevent mortality and morbidity.

### Limitations

A detailed biochemical analysis of all participants was performed, but the lack of sex hormone–binding globulin levels and free testosterone values represents a limitation of our study. Another limitation is that the severity of ED was determined using the IIEF-5 scores, rather than a more objective method, such as penile artery ultrasound. Penile Doppler ultrasound would have provided more accurate results, but due to the invasive nature of the procedure and ethical issues, we were not able to perform this examination within the study setting. Also, patients receiving treatment for ED were not evaluated in our study, and therefore these markers could not be used to assess the response to treatment in this study design.

## Conclusion

Atherogenic indices and the TyG index can be used as inexpensive and practical markers to predict the severity of arteriogenic ED. According to our study, especially the relationship of AIP and TyG index with ED is important in urology practice. We consider that these 2 parameters can be used in the diagnosis and follow-up of ED. However, our data should be supported by more detailed, large-scale, prospective controlled studies. There is also a need for further studies to evaluate post-treatment levels of atherogenic indices and the TyG index and pretreatment vs post-treatment comparisons. In this way, these markers can also be used as predictors of treatment response.

## Funding

None declared.

## Conflicts of interest

The authors declare that they have no conflict of interest.

## Data availability

Data of patients and statistical analysis data used to support the findings of this study are available from the corresponding author upon request.
